# Perceiving Time Differences When You Should Not: Applying the El Greco Fallacy to Hypnotic Time Distortions

**DOI:** 10.3389/fpsyg.2016.01309

**Published:** 2016-08-30

**Authors:** Jean-Rémy Martin, Jérôme Sackur, Hernan Anlló, Peter Naish, Zoltan Dienes

**Affiliations:** ^1^School of Psychology, University of SussexBrighton, UK; ^2^École des Hautes Études en Sciences Sociales, Laboratoire de Sciences Cognitives et Psycholinguistique (CNRS/ENS/EHESS), Département d’Études Cognitives, Paris Sciences et Lettres Research UniversityParis, France; ^3^Centre de Recherche InterdisciplinaireParis, France; ^4^Department of Psychology, The Open UniversityMilton Keynes, UK; ^5^Sackler Centre for Consciousness Science, University of SussexBrighton, UK

**Keywords:** time perception, time distortion, greco fallacy, hypnosis, suggestion

## Abstract

The way we experience and estimate time – subjective time – does not systematically correspond to objective time (the physical duration of an event). Many factors can influence subjective time and lead to mental dilation or compression of objective time. The emotional valence of stimuli or the levels of attention or expectancy are known to modulate subjective time even though objective time is constant. Hypnosis too is known to alter people’s perception of time. However, it is not known whether hypnotic time distortions are intrinsic perceptual effects, based for example on the changing rate of an internal clock, or rather the result of a response to demand characteristics. Here we distinguished the theories using the logic of the El Greco fallacy. When participants initially had to compare the duration of two successive events —with the same duration — while in “trance,” they responded that the second event was on average longer than the first event. As both events were estimated in “trance,” if hypnosis had impacted on an internal clock, they should have been affected to the same extent. Conversely, when only the first event was in “trance,” there was no difference in perceived duration. The findings conform to an El Greco fallacy effect and challenge theories of hypnotic time distortion arguing that “trance” itself changes subjective time.

## Introduction

The way we experience and estimate time – subjective time – does not systematically correspond to objective time (the physical duration of an event). Many factors can influence subjective time and lead to mental dilation or compression of objective time. As an illustration, the emotional valence of stimuli (Droit-Volet and Gil, 2009) or the level of attention or expectancy (Coull et al., 2004; Tse et al., 2004) are known to modulate subjective time even though objective time is constant (for a review, see e.g., Grondin, 2010). Dilation leads to over-evaluation of objective time and compression leads to under-evaluation of objective time. The fact that time perception is so “malleable” is probably due to the fact that there is no “absolute time” ability analogous, for instance, to absolute pitch (van Wassenhove, 2009).

In this respect, some evidence suggests that hypnosis is able to distort time perception (Bowers, 1979; Bowers and Brenneman, 1979; St Jean et al., 1994; Naish, 2001, 2006). More precisely, hypnotic induction can compress time perception in the case of what Naish (2001) calls *retrospective duration judgements* (i.e., judgments made after the duration is up) (Bowers and Brenneman, 1979; Naish, 2001), and dilate it in the case of *prospective duration judgments* (i.e., the participant indicates when she/he thinks that a specific duration is now up) (Naish, 2001, 2003). Naish (2006) proposed that the alleged hypnotic state specifically slows down the internal clock; a putative dedicated central mechanism underlying time perception (Creelman, 1962; Treisman, 1963; Rammsayer and Ulrich, 2001). In this approach, time judgments are based on an internal clock composed of a pacemaker emitting pulses that are accumulated in a counter. The number of counted pulses determines the length of the perceived duration. Therefore, if hypnosis slows the internal clock (e.g., the rate of pulses emitted by the pacemaker), time perception should indeed be compressed (under-evaluated) in the case of retrospective judgments (Naish, 2006). This could also explain over-evaluation in the case of prospective judgments as defined by Naish (2001, 2006): since the clock is running slower, participants wait longer than objective time before reporting that the duration is up (i.e., if 2 min seem like one, then the participant will wait 4 min before reporting that 2 min have passed). We will refer to this hypothesis as the *Slow Clock Theory.*

Nonetheless, considering that the putative hypnotic state distorts time perception raises a number of issues. Crucially, current available evidence does not allow us to decide whether hypnotic time distortions are intrinsic perceptual effects based on “trance” itself or rather the result of expectations based on the participants’ understanding of demand characteristics (when participants are explicitly asked to perform temporal judgments).

In the present study, we investigated this issue using the logic of the *El Greco fallacy* (Firestone, 2013; Firestone and Scholl, 2014). El Greco was an artist from the Spanish Renaissance well known for having painted particularly elongated figures. It has been conjectured that El Greco might have suffered from strong astigmatism, perceptually distorting the world as if vertically stretched out. At first glance, this might explain why El Greco painted elongated figures; simply because he saw the word stretched out. However, this explanation is fallacious: if El Greco perceived the world vertically stretched out, then that is also how he would see the canvas on which he painted his figures. Therefore, “the real-world distortions he experienced would never have transferred to his reproductions” (Firestone and Scholl, 2014, p. 39). Based on this logic, Firestone and Scholl (2014) developed a method to decide whether the effect of different experimental conditions on perceptual decision reflects truly perceptual effects or demand characteristics (Orne, 1959).

Here, we use the logic of the *El Greco fallacy* in order to test whether the potential effects of hypnosis on time perception are truly perceptual or not. We designed a duration comparison task in which a standard and a target disk presented successively on the screen had the *same* duration. Participants were asked to evaluate whether the duration of the target disk was Shorter or Longer than the standard disk in different conditions; participants were not told that both disks had the same duration. In one condition (Greco Condition), participants performed the task while in “trance” during the whole trial, in a second condition participants were in a normal, “alert state” throughout the trial (Control Condition) and, finally, in the third condition, participants were in a normal “alert state” before the standard disk but in “trance” before the target disk was displayed (Non-Greco Condition).

In the Control Condition, participants should judge the target disk to be Longer than the standard disk 50% of the time (as both disks have the same duration). Similarly, during the Greco Condition participants should also judge the target disk to be Longer than the standard disk 50% of the time, because participants are in “trance” both before the standard disk *and* the target disk. Even if the putative Slow Clock is in operation, the duration of both disks should be affected to the same extent, so any bias in judgment would be analogous to a Greco fallacy effect. Crucially, however, in the Non-Greco Condition, participants should judge the target disk to be Shorter more often than Longer, as compared to the standard disk (according to the Slow Clock Theory). Participants being in “trance” before the target disk but not before the standard disk, only the duration of the second (target) disk should be distorted.

## Materials and Methods

### Participants

Twenty-one subjects (12 females, Mean Age = 24.5, *SD* = 4.2) scoring 9–11 (*M* = 10, *SD* = 0.7) were recruited as high hypnotizable subjects from a larger sample screened with the French version of the Harvard Group Scale of Hypnotic Susceptibility, Form A (Shor and Orne, 1962; Anlló et al., in press). Each subject was paid 10€ for participation, the whole experiment lasting approximately 1 h. Subjects had normal or corrected-to-normal vision.

Bayesian analyses were used to assess sensitivity. In particular, we used Bayesian analyses to indicate the strength of evidence for H1 versus H0; the measure of evidence is valid no matter what the stopping rule is (Rouder, 2014; Schoenbrodt et al., 2015). Participants were recruited until the Bayes factor for *post hoc* analyses following ANOVA were about >3 or <1/3. The stopping rule undermines the meaning of frequentist statistics; thus, interpretation will be made with respect to the Bayesian statistics.

Written informed consents were obtained from each participant and the experiment was conducted in a properly ethical manner in agreement with the Declaration of Helsinki (2008). The present study was approved by the Ethics Committee of the Université Paris Descartes (Paris 5, France).

### Experimental Setup and Apparatus

The experiment was conducted in a quiet experimental room. Stimuli were delivered by a MacBook Pro, processor 2.53 GHz, Intel Core i5. All stimuli were displayed using Matlab (MathWorks Inc. R2009b) with the Psychophysics Toolbox (Brainard, 1997).

### Hypnotic Procedure

We used a 9-min induction, based on the Harvard Group Scale of Hypnotic Susceptibility, Form A (Shor and Orne, 1962; Anlló et al., in press). At the end of the induction, the subject was administered the suggestion that every time the word *Trance* was displayed on the screen she would enter into hypnotic “trance,” and every time the word *Normal* was displayed she would stay or come back into a normal state of consciousness. The exact wording of the suggestion was as follows (it was administered in French):

When the count is finished and you are back to your normal waking state, you will perform an experiment on the computer. However, listen carefully to what I am going to tell you. In each trial of the experiment, the word *trance* or the word *normal* will be displayed on the screen… and each time you see the word *trance* I want you to slip into the hypnotic state in which you are now, but keeping your eyes open. Each time the word *trance* is displayed, you will be as deeply hypnotized as you are now, and you will be perfectly able to do what you are being asked to do. Conversely, each time you see the word *normal* you will stay or come back into a perfectly normal state of wakefulness. Now, by the time I reach “five” you will open your eyes, but you will not be fully awake. When I get to “one,” you will be fully alert, in your normal state of wakefulness. Remember that each time you see the word *trance* on the screen you will sleep into a deep trance hypnotic state while keeping your eyes open, and each time you see the word *normal* you will stay or come back into a perfectly normal state of wakefulness.

After the suggestion, participants were asked to rate the depth of their hypnotic “trance” during the induction procedure on a scale from 0 (no trance) to 5 (very deep trance). In addition, before starting the main task, the words NORMAL and TRANCE were displayed alternately on the screen three times each, and subjects were asked to rate the mean depth of their hypnotic “trance” following each word on the same scale as above.

Finally, once the tasks were completed, participants received a de-induction procedure whereby the suggestion was canceled. A debriefing followed and concluded the experiment.

### Stimuli and Experimental Design

**FIGURE 1:**
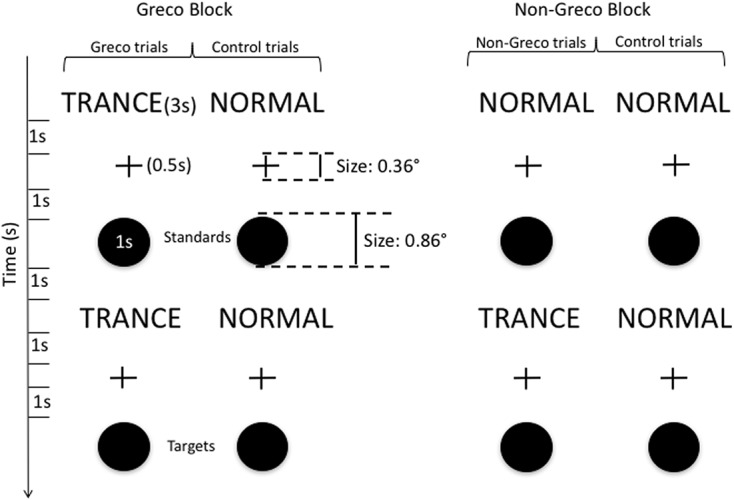
**Stimuli and delays.** The figure describes the physical properties of stimuli, their duration, and the delays between them. In every trial, a first word (NORMAL or TRANCE in the Greco Block and NORMAL in the Non-Greco Block) was displayed for 3 s at the centre of the screen. After a delay of 1 s, a fixation cross was displayed at the centre of the screen for 0.5 s. One second after the disappearance of the cross, the standard disk (1 s) was displayed at the centre of the screen. One second after, the second word (TRANCE or NORMAL) was displayed. Then the fixation cross and the target disk with identical delays and durations were displayed. One second after the disappearance of the target disk, participants had to answer the duration comparison question (not shown in the figure). Once they answered the question, the word NORMAL was displayed and then participants answered successively the two subjective scales appearing on the screen after the word disappeared. Subjective scales asked participants to evaluate the depth of their “trance” [from 0 (no trance) to 5 (very deep trance)] before the first disk (first scale) and before the second disk (second disk) (not shown in the figure, see main text).

**Figure 1** shows the exact delays between the different stimuli and their durations. Subjects were seated at about 80 cm from the screen. In every trial, a standard black disk (diameter = 1.2 cm/0.86°) with a duration of 1 s, preceded by a fixation cross (duration = 0.5 s, width and height size = 0.5 cm/0.36°), was first displayed at the centre of the screen. After some delay (see below), a second identical target disk with the same duration, still preceded by a fixation cross, was displayed. Participants had to decide whether the duration of the target disk was Shorter (by pressing the left arrow key) or Longer (by pressing the right arrow key) than the first disk. Participants were not told that both disks had the same duration.

The experiment included three experimental conditions. In *Control trials* (Control Condition), the first and second disk were preceded by the word NORMAL displayed at the centre of the screen (duration = 3 s), in *Greco trials* (Greco Condition), the first and second disk were preceded by the word TRANCE, finally, in *Non-Greco trials* (Non-Greco Condition), the first disk was preceded by the word NORMAL and the second disk was preceded by the word TRANCE (the inter-stimulus interval (ISI) between words and disks was 2.5 s, which includes the fixation cross duration, namely 0.5 s).

In every trial and condition, once participants answered the duration comparison question, the word NORMAL was displayed at the centre of the screen, and two successive subjective scales appeared on the screen asking participants to evaluate the depth of their “trance” before the first disk (first scale) and before the second disk (second scale), from 0 (no trance) to 5 (very deep trance).

The experiment comprised two blocks: the *Greco Block* was composed of Greco trials and Control trials, the *Non-Greco Block* was composed of Non-Greco trials and Control trials (the presentation of trials was randomized). There were 10 trials for each condition; 40 trials in total. Half of participants were presented with the Greco Block first and half of participants performed the Non-Greco Block first (subjects were assigned to order in an alternating way). The two blocks were necessary in order to avoid direct comparison between trial types, especially between Greco trials and Non-Greco trials that may have led participants to understand how they should respond to the Greco trials. Thus, Greco and Non-Greco trials were separately mixed with their own Control trials (referred to as Control Greco trials and Control Non-Greco trials, respectively).

Between the first and second block, participants were administered a short re-induction procedure in which the suggestion was administered again.

### Statistical Analysis

We report Bayes factors, *B*, to assess strength of evidence for the alternative hypothesis, H1, compared to the null hypothesis, H0 (Morey et al., 2016) for all one degree of freedom effects. A *B* of 3 or above indicates “substantial evidence” for the alternative rather than the null hypothesis, to use the term of Jeffreys (1939) – “substantial” in the sense of evidence starting to be worth taking note of (Lee and Wagenmakers, 2013). By symmetry, a *B* of 1/3 or below indicates substantial evidence for the null rather than alternative hypothesis. Thus, a *B* between 3 and 1/3 indicates data insensitivity for distinguishing the alternative and null hypotheses.

Regarding the suggestion checks carried out after the induction phase, and also the “trance” depth ratings taken on each trial, since a scale of 0–5 was used, the maximum difference in ratings between the word NORMAL and the word TRANCE could be 5. Hence, we used a uniform distribution from 0 to 5, written *B*_U[0,5]_.

According to the Slow Clock Theory (i.e., that hypnosis slows the internal clock), the percentage of Longer responses should be 50 in the Greco condition and the percentage of Longer responses should be lower in the Non-Greco Condition than in the Greco Condition. In other words, the difference between the percentage of Longer responses and the percentage of Shorter responses should be zero in the Greco condition and should be negative in the Non-Greco condition. Therefore, the biggest difference that could be expected is 50% in any direction. As smaller differences are more likely than bigger ones, we modeled the alternative hypothesis (H1) using a normal distribution centered on zero with a standard deviation (SD) of 25%, referred to as *B*_N(0,25)_ (See Dienes, 2014, 2015, for this notation)^1^. For simplicity, we used the same H1 for every test. In addition, in cases where the Slow Clock Theory predicts specific direction effects as in the Non-Greco Condition (fewer Longer responses than Shorter ones) we used a half-normal distribution with a mean of 0 and a SD of 25%, referred to as *B*_H(0,25)._

### Trance Depth Differences after the Induction and before the First Block

Participants had to rate the depth of their hypnotic “trance” three times: for the induction, the word TRANCE and the word NORMAL (See *Hypnotic Procedure*). We conducted paired *t-tests* which gave evidence that our posthypnotic suggestion had the desired effect: The word TRANCE (*M* = 2.57, *SD* = 0.87) produced a deeper “trance” than the word NORMAL (*M* = 0.42, *SD* = 0.67, *t*_20_ = 10.78, *p* < 0.001; *B*_U[0,5]_ = 6.07 × 10^21^).

In addition, we checked to what extent the induction (*M* = 3.71, *SD* = 0.46) produced a deeper “trance” than the words NORMAL and TRANCE: Induction *versus* the word NORMAL (*t*_20_ = 15.74, *p* < 0.001; *B*_U[0,5]_ = 2.08 × 10^52^), Induction *versus* the word TRANCE (*t*_20_ = 5.43, *p* < 0.001; *B*_U[0,5]_ = 2.6 × 10^5^)_._ In sum, the induction produced a deeper “trance” than either word.

### Trance Depth Differences between Subjective Scales

We performed another series of paired *t*-tests on “trance” depth ratings between the two subjective ratings on each trial, for each experimental condition (Control, Greco and Non-Greco Condition) in order to determine whether the posthypnotic suggestion produced the changes in “trance” depth required throughout the experiment.

For the group receiving the Greco Block first, the mean differences between the two subjective ratings were 0.04 (*SD* = 0.33) in the Control Greco Condition (*t*_10_ = 0.45, *p* = 0.66; *B*_U[0,5]_ = 0.04); 0.05 (*SD* = 0.31) in the Control Non-Greco Condition (*t*_10_ = 0.58, *p* = 0.57; *B*_U[0,5]_ = 0.04); 2.26 (*SD* = 1.00) in the Non-Greco Condition (*t*_10_ = 7.43, *p* < 0.001; *B*_U[0,5]_ = 6.5 × 10^7^); and 0.40 (*SD* = 0.39) in the Greco Condition (*t*_10_ = 3.47, *p* = 0.006; *B*_U[0,5]_ = 7.40).

For the group presented with the Non-Greco Block first, the mean differences were 0.08 (*SD* = 0.18) in the Control Greco Condition (*t*_9_ = 1.35, *p* = 0.21; *B*_U[0,5]_ = 0.06); 0.03 (*SD* = 0.16), in the Control Non-Greco Condition (*t*_9_ = 0.58, *p* = 0.57; *B*_U[0,5]_ = 0.02); 1.96 (*SD* = 0.76) in the Non-Greco Condition (*t*_9_ = 8.07, *p* < 0.001; *B*_U[0,5]_ = 1.2× 10^9^); and 0.94 (*SD* = 0.62) in the Greco Condition (*t*_9_ = 4.72, *p* = 0.001 ; *B*_U[0,5]_ = 488.53).

In sum, analyses showed that our posthypnotic suggestion produced the desired effect. More precisely, in the Non-Greco Condition, the word TRANCE produced a deeper “trance” than the word NORMAL, and in the Control Condition there were no differences in “trance” depth between the two words NORMAL. In the Greco Condition, the second word TRANCE produced a slightly deeper “trance” than the first word TRANCE, but it was small.

### Effects of the Posthypnotic Suggestion on Time Judgments

**FIGURE 2:**
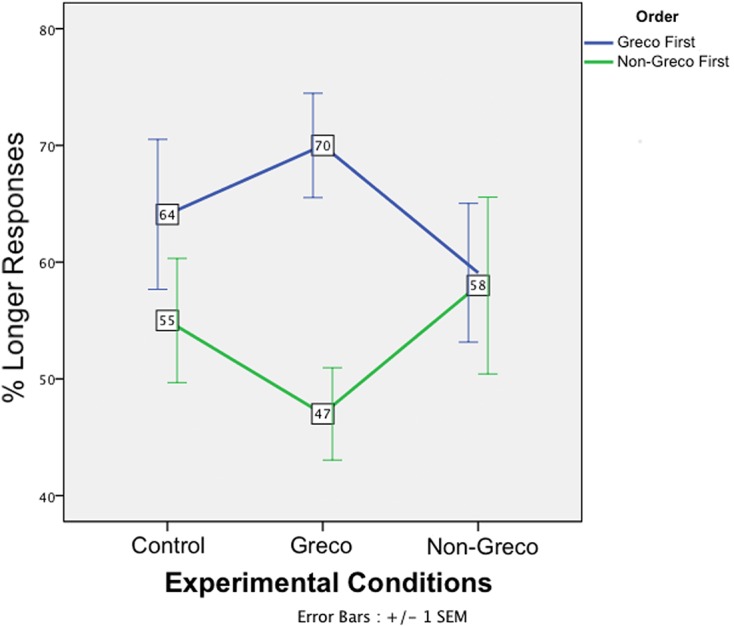
**Effects of the posthypnotic suggestion on time judgments.** The graph shows the mean percentage of Longer responses according to Experimental Conditions (Control, Greco and Non-Greco types of trials) and participants presented with the Greco Block first (blue) and participants presented with the Non-Greco Block first (green). Error bars indicate 1 ± SEM.

**Figure 2** shows the mean percentage of Longer responses according to condition (Control, Greco and Non-Greco types of trials) and block order (Greco first or Non-Greco first).

In order to evaluate the effect of the different experimental conditions (types of trials), we performed a mixed-design ANOVA with the percentage of Longer responses as the dependant variable, Experimental Condition (three levels: Greco, Non-Greco and Control Condition) as the within-subjects factor, and Block Order (Greco Block first or Non-Greco Block First) as the between-subjects factor. Note that the level “Control Condition” consists in the two control conditions (the one present in the Greco Block and the one present in the Non-Greco Block) merged together. A Bayes factor on the percentage of Longer responses between the two control conditions showed evidence for no difference between them (Mean difference = 0.48%, *t*_20_ = 0.089, *p* = 0.930; *B*_N[0,25]_ = 0.21). Therefore, we averaged the percentage of Longer responses over the two control conditions for every participant.

The ANOVA showed no significant main effect of the within-subjects factor *Experimental Condition* (*F*_2,38_ = 0.023, *p* = 0.98). There was little evidence for the main effect of *Block Order* (*F*_1,19_ = 4.84, *p* = 0.040, B_N[0,25]_ = 1.03). The interaction Experimental Condition^∗^Block Order was not significant (*F*_2,38_ = 2.00, *p* = 0.149).

Finally, we conducted single *t*-tests against 50% within each group for each condition, namely six single *t*-tests (with Bonferroni correction).

The Slow Clock Theory predicts that whatever the group (Greco first or Non-Greco first) the percentage of Longer responses should be 50% in both the Control and Greco Conditions, and less than 50% in the Non-Greco Condition. It is less easy to predict the pattern of responses, if driven by demand characteristics. Presumably, this would also predict 50% of Longer responses in the Control Condition, but in contrast to the Slow Clock Theory, the Greco Condition may be treated similarly to the non-Greco. There are two unknown factors here. First, it is not clear whether participants would expect a time contraction or dilation effect, so the percentage of Longer responses may be greater or less than 50%. Second, we do not know how sophisticated the interpretation of demand characteristics might be (i.e., whether the ‘trap’ in the Greco condition is recognized). Plausibly, an appreciation of the fallacy implicit in the Greco condition may be expected to depend on whether the non-Greco condition had already been experienced. Thus, an interaction with order of presentation would be the defining feature of a demand characteristic account. If both a slow clock and demand characteristics were operating, then a more complex pattern of results may emerge.

For the group presented with the Greco Block first, the mean differences from 50% were 14.09% in the Control Condition (*t*_10_ = 2.19, *p* = 0.053; *B*_N[0,25]_ = 1.40); 9.09% in the Non-Greco Condition (*t*_10_ = 1.52, *p* = 0.157; *B*_N[0,25]_ = 0.58, *B*_H[0,25]_ = 0.13); and 20% in the Greco Condition (*t*_10_ = 4.47, *p* = 0.001; *B*_N[0,25]_ = 162.90).

For the group presented with the Non-Greco Block first, the mean differences from 50% were 5% in the Control Condition (*t*_9_ = 0.94, *p* = 0.37; *B*_N[0,25]_ = 0.33); 8% in the Non-Greco Condition (*t*_9_ = 1.05, *p* = 0.3; *B*_N[0,25]_ = 0.48; *B*_H[0,25]_ = 0.21); and -3% in the Greco Condition (*t*_9_ = -0.76, *p* = 0.46; *B*_N[0,25]_ = 0.23).

In sum, the Non-Greco Condition provided evidence for H0 and against hypnotic “trance” slowing the internal clock. Conversely the Greco Condition, when it came first, provided a demonstration of the El Greco fallacy, and hence evidence for the operation of demand characteristics.

## Discussion

The present study investigated the nature of time distortions in hypnosis. It has been hypothesized that hypnosis affects time perception at the processing level in slowing down the putative internal clock sustaining our ability to estimate time (Naish, 2001, 2003, 2006). We referred to this hypothesis as the Slow Clock Theory. However, experimental designs used up to now to study time estimation in hypnosis cannot tell us whether hypnotic time distortion truly reflects alterations at a perceptual (process) level, or pure demand characteristics. Based on the logic of the El Greco fallacy, we designed an experiment in order to disentangle this issue.

Under the Slow Clock Theory, the only situation in which time distortion should have been registered was the Non-Greco Condition. Specifically, participants should have answered Shorter more often than Longer. Contrary to expectations, Bayes factors indicate that, in this condition, the percentage of Longer responses was *not* different from 50%. One might argue, however, that participants were insufficiently hypnotized to produce the timing changes. The subjective ratings in this experiment indicated rather modest levels of hypnotic response and there is evidence that the magnitude of the effect is dependent upon susceptibility (see Naish, 2014, for a discussion of the issue). Without available psychometric functions between hypnotic depth and time distortion in the current literature, it is nonetheless difficult to infer that our participants were not sufficiently hypnotized to produce the timing changes. Future studies are needed to disentangle this issue.

In addition, the Greco trials produced surprisingly contrasting results to the above. While the Slow Clock Theory predicts no time distortion in this condition (target and standard both evaluated under “trance”) the participants produced responses that did differ from the ideal 50% rate, showing that time changes were possible with the current design. In other words, participants exhibited an El Greco fallacy effect, although they made more, rather than fewer, Longer judgments, as if the putative clock had sped up between the standard and target presentations. However, this effect was confined to the condition in which the Greco condition was performed first.

An attempt at a partial explanation for these findings might propose that the slowing effect produced by hypnosis takes some time to become established. In the Non-Greco trials, the rapid switch from “Normal” for the standard, to “Trance” for the target, allowed insufficient time for the inner clock to slow, and as a consequence there was no tendency to produce more Shorter judgments. This would explain why the results for this condition were close to 50%. For the Greco condition, the target was presented shortly after the participant was instructed to enter a “trance,” so the clock would still be “ticking” at close to its normal rate. However, by the time the target was presented some slowing would have occurred, so the target duration would appear to differ from the standard. The problem for this account is that the targets would seem shorter than the standards, whereas in the data actually obtained the reverse was true. Moreover, a ‘not-yet-settled-down’ account should be equally applicable, in whatever order the blocks were encountered; in our results the timing disparity was apparent only when the Greco condition was presented first. Finally, note that the “trance state” did not decay over time (see Supplementary Figures S1 and S2) so that it cannot be argued that participants were not in “trance” at some point while viewing the comparison stimulus, for instance.

If a specific hypnosis-induced change is an implausible cause of time distortion, then the alternative is that beliefs and expectations about hypnosis (so-called demand characteristics) were the influencing factor. This is certainly applicable across the whole spectrum of hypnotic responding. Beliefs about hypnosis are widespread and expectations can frequently be gleaned from the words of the hypnotist. That this is the case was demonstrated by Orne (1959). He reported that people of low susceptibility, when told to act as they believe a hypnotized person would act, are able to do so sufficiently convincingly as to deceive an expert into believing them to be deeply hypnotized. Using ‘simulators’ in this way has become a standard means of disentangling effects produced by demand characteristics from those that may be an intrinsic result of hypnosis (but see Barber, 1969 for a critique of simulators as a control group. To the extent that simulators are explicitly asked not to try to experience the subjective effects associated with hypnosis, their expectations and task-demands are rather different from non-simulators. In addition, simulators are drawn from the population of lows for which expectations about hypnosis are potentially different from highs’ expectations). Mozenter and Kurtz (1992) compared highs to simulators (and non-simulator lows) in a verbal estimation task in which participants had to report verbally the duration of various time intervals (30, 60, 120, and 240 s) filled with white noise. Results are complex; for some intervals highs overestimated time and for at least one interval simulators showed a bias but in the opposite direction (underestimation). Overall this study is not conclusive as to whether hypnosis affects time perception at a process level or not, as it counts both against the Slow Clock Theory (time underestimation) and an explanation in terms of pure demand characteristics.

We propose that in the present study, participants answered both according to their beliefs about the way hypnosis affects time perception and with respect to the experimental manipulations in force. During post-experimental debriefing, we asked participants how they thought hypnosis affected time perception. Eighty percent (17 out 21) replied that hypnosis slows time. When asked to clarify the statement, they said that a stimulus of a certain duration would appear retrospectively *longer* if time were slowed down. This is exactly the effect observed in the data, so it can be argued that these participants were indeed producing the effects that they believed to be applicable to hypnosis. Against this interpretation, it might be argued that participants truly experienced some time distortion and then later, when asked, report holding a belief consistent with their experience. The existence of time distortions is not something that is nonetheless evident from the experience itself. Further, participants did not experience time distortions in the current experiment; as shown by the absence of a time distortion effect in the Non-Greco Condition. In order to strengthen the claim that participants followed their beliefs about the way hypnosis affects time perception in reporting their time judgments under the different conditions, future research could obtain belief reports prior to the task or explicitly manipulate pre-task beliefs about time perception during hypnosis (through informing participants that hypnosis speeds up or slows down the subjective experience of time), or, again, ask an independent group of subjects^2^.

Two questions remain unanswered: why the effect discussed above occurred only when the El Greco block was encountered first, and why no effect was observed in the Non-Greco block. A possible explanation concerns the nature of the Non-Greco block. It was a relatively complex condition, and the switching in and out of a resource-demanding task (i.e., responding to the hypnotic suggestion, Wyzenbeek and Bryant, 2012) may have made it harder for participants to recognize what might be expected of them. Nevertheless, having experienced this task first may have left participants sufficiently well informed that, when they encountered the El Greco block they recognized the illogicality of making Longer judgements when the standard had also been viewed in hypnosis.

In sum, we found changes in time perception concomitant with hypnotic “trance” consistent with demand characteristics but not with a putative change in the internal clock brought about by trance. “Trance” may well be experienced in a compelling way; but it may have the felt qualities simply that participants expect of it.

A general consideration for all time perception research is therefore the extent to which estimates of time may be based on a time-specific mechanism like an accumulator, or rather on expectations determined by demand characteristics. In general, the results indicate that research into time perception may easily fall foul of demand characteristics when trying to characterize domain-specific mechanisms.

## Author Contributions

J-RM, conceived the experiment and its design, analyzed data, performed the experiment, and wrote up the manuscript. JS, analyzed data, wrote up the manuscript. HA, screened the larger pool of participants from which the pool of the current experiment has been drawn and reread the manuscript. PN, contributed to interpret data, and wrote up the manuscript. ZD, contributed to the design of the experiment, analyzed data, and wrote up the manuscript.

## Conflict of Interest Statement

The authors declare that the research was conducted in the absence of any commercial or financial relationships that could be construed as a potential conflict of interest.

## Footnotes

1 In order to test whether this model of the alternative hypothesis was justified, we conducted the following calibration experiment: Naish (2001) found that hypnosis compresses retrospective time estimations for both long periods (many minutes) and short periods (a few seconds). In the first case, time estimations under hypnosis were 68% of time estimations out of hypnosis; and, for the second case, the time period more similar to ours, 81%. Therefore, we designed a comparison task where the standard was always 1s and the target either 1s too or 0.8 s (50 trials in both target conditions). Ten subjects (Mean Age = 30.2, *SD* = 4.75), answered *Longer* or *Shorter* for every trial by means of a key press. The mean proportion of Longer responses was 59.2 % (*SD* = 18.7) in the 1 s-target condition and 20.8% (*SD* = 15.6) in the 0.8 s-target condition. For comparison, our model of H1 for the main experiment assumed the rough order of scale for time distortion was to produce longer estimates 75% of the time for subjectively lengthened intervals. In sum, this calibration experiment justifies the values used to model the H1 (*B*_N[0,25]_) for BFs assessing the strength of evidence when comparing the proportion of Longer responses between the different experimental conditions.

2 For converging evidence, we asked an independent group of subjects (unselected for hypnotisability) the following, by means of a short online survey: “Intuitively, do you think that hypnosis slows down of speeds up time perception? For instance, suppose that I display to you a sound for 5 s and then that I ask you to evaluate its duration once it is up. If hypnotized, do you think you would evaluate the sound as shorter or longer than it really is? (translated from the French version given to participants).” Seventy-six percent of subjects (*N* = 17, 9 females, Mean Age = 33.5; *SD* = 5.7) said they would evaluate the sound as *longer*. This result is in line with our post-experimental debriefing, consistent with the conjecture that a common pre-existing belief is that hypnotic trance would make intervals seem longer.
