# Supplementary figures and images for: Perceiving Time Differences When You Should Not: Applying the El Greco Fallacy to Hypnotic Time Distortions
Source: Front Psychol. 2016 Aug 30;7:1309. doi: 10.3389/fpsyg.2016.01309 (PMC5004410; doi:10.3389/fpsyg.2016.01309)

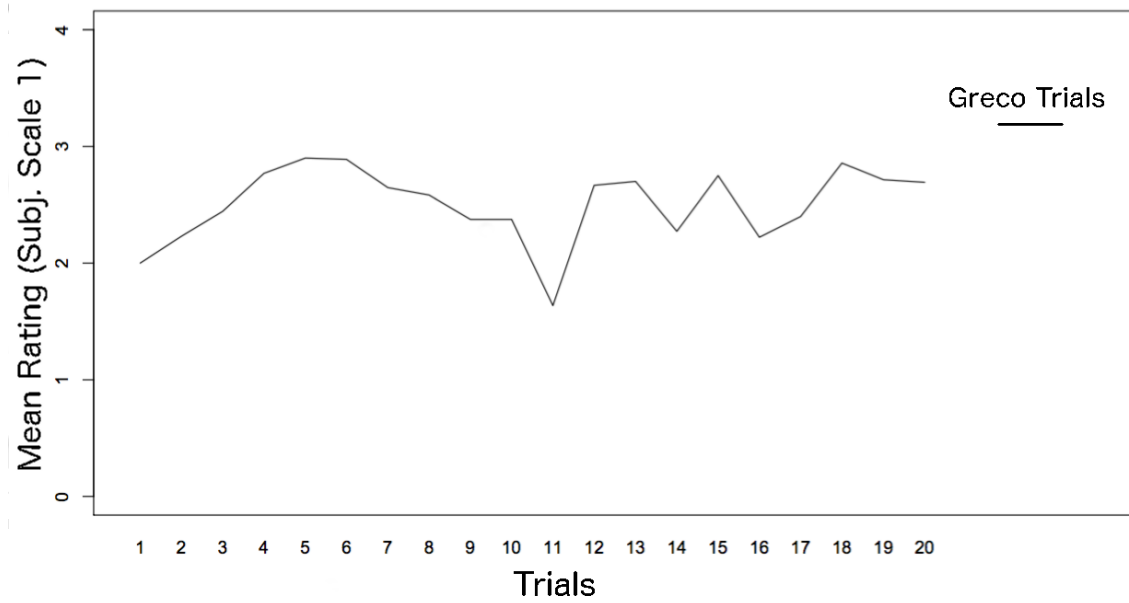

**S1.** Mean trance rating in the Greco Condition before the first disk (Subjective Scale 1) by trial.

Supplement: Supplementary file 1 [file Image_1.PDF]

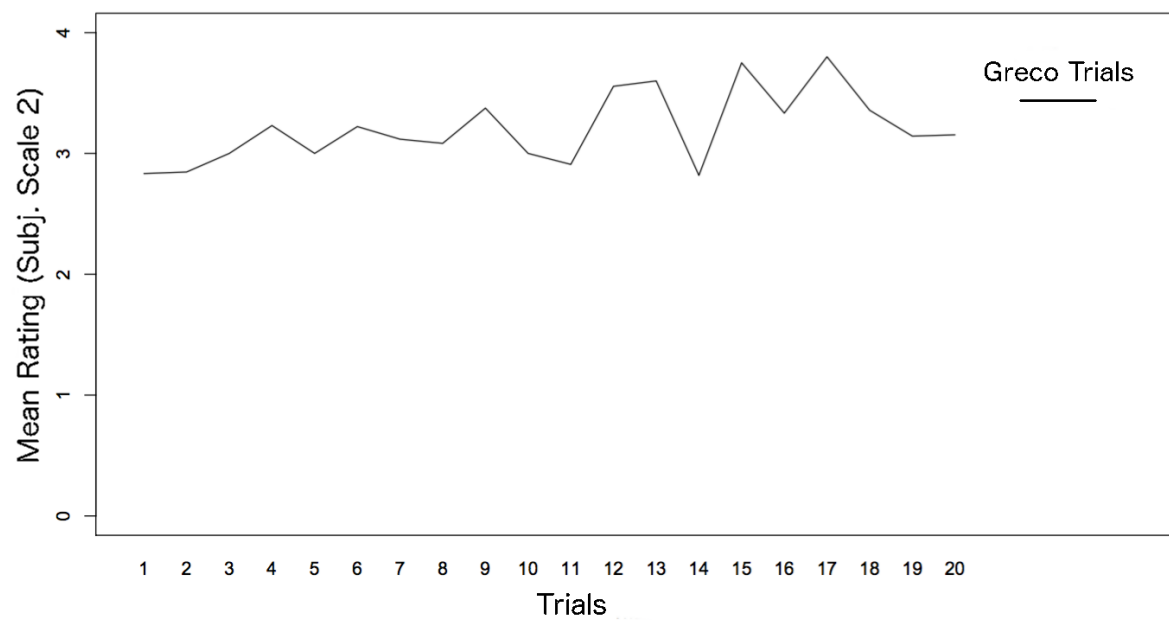

S2. Mean trance rating in the Greco Condition before the second disk (Subjective Scale 2) by trial.

Supplement: Supplementary file 2 [file Image_2.PDF]
